# In vitro protective effects of *Paeonia officinalis* var. *mascula* callus extract on human keratinocytes

**DOI:** 10.1038/s41598-020-76169-0

**Published:** 2020-11-05

**Authors:** Sophia Letsiou, Artemis Bakea, Anna Holefors, Jadwiga Rembiesa, Eleni Spanidi, Konstantinos Gardikis

**Affiliations:** 1 Laboratory of Biochemistry, Research and Development Department, APIVITA S.A., Industrial Park of Markopoulo Mesogaias, Markopoulo Attiki, 19003 Athens, Greece; 2 In Vitro Plant-Tech AB, Geijersg 4B, 21618 Limhamn, Sweden

**Keywords:** Biotechnology, Transcriptomics

## Abstract

Natural ingredients have been used to improve the state of health in humans. The genus *Paeonia* has been studied only limited yet it’s reported to have many activities such as antioxidant and anti-inflammatory. To this context, here we focused on an endemic Paeonia species in Attica*.* This study aims to present the development of the *Paeonia officinalis* var. *mascula* callus extract and its pleiotropic bioactivity on human primary keratinocytes exploring its potential application as an active agent in skin-related products. This extract showed a high scavenging activity with high phenolic content and an interesting metabolic profile. At a molecular level, the study on the transcript accumulation of genes revealed that this extract exhibits in vitro skin-related protection properties by mediating mitochondrial energy, cell proliferation, immune and inflammatory response and positively regulates genes involved in epidermal and in stratum corneum function. Besides, the extract is proven not skin irritant on reconstructed human skin model. These findings indicate that the specific *P. officinalis* var. *mascula* extract possesses significant in vitro protection activity on human epidermis and provides new insights into its beneficial role in skin confirming that the advent of biotechnology contribution the past few decades.

## Introduction

From time immemorial since our days, plant-based products are used as effective ingredients for many purposes such as pharmacology, nutrition, or agrochemistry. Recently, the advent of biotechnology offers a plethora of methods to facilitate the use of plants in different products enhancing as well as preserving their efficacy as biotechnological in vitro methods aim to counteract the extension of rare natural species. Moreover, the use of natural extracts in skin care products is constantly increasing due to their protective properties^1^. To this context, here we present bioactivity assessments for the extract of *Paeonia officinalis* var. *mascula*.

In general, the genus *Paeonia* (*Paeoniaceae*) which has three sections (Moutan, Oneapia and Paeonia), has been studied only limited^2^. Specifically, from the 262 compounds that were identified in different parts of *Paeonia* only several anthocyanins were reported in *Paeonia officinalis* (*P. officinalis*) flowers and one (1,2,3,6-tetra-O-galloyl-d-glucose) in its roots^2^. A large repertoire of bioactive substances were found in different *Paeonia* spp. that are responsible for their biological and pharmacological activities such as, in treatment for epilepsy, liver diseases and many other disorders^3^. Furthermore, it has been reported that *Paeonia* plants have many activities such as antioxidant, anti-inflammatory, anti-microbial, immune system modulation^4–7^. Besides the above, some *Paeonia* spp. such as *P. lactiflora* and *P. suruticosa* have been considered as a rich nutritional source of polyunsaturated oil and proteins^8^.

Among the 25 species of the section *Paeonia* that is distributed widely in Eurasia, *Paeonia clusii* and *Paeonia parnassica*, are endemic in Greece. The Greek *Paeonia* taxa, *Paeonia officinalis* var. *mascula* L. has been studied only limited. Specifically, it has been reported that the roots of Greek *Paeonia* taxa has wound healing properties^9^. Another study reported that *Paeonia officinalis* var. *mascula* (*P. officinalis* var. *mascula*) extract has strong skin whitening properties since its aerial parts was proven to provide a strong tyrosinase inhibition^10^.

Considering, the uniqueness, the rarity and the potential efficacy of *P. officinalis* var. *mascula* in skin, here we present the development of *Paeonia officinalis* var. *mascula* callus extract (*POCE*), for the very first time, as well as its in vitro biological effects in human primary keratinocytes with the aim to be used as an active ingredient in the development of skin-related products. To elucidate the molecular mechanism of *POCE* bioactivity, we studied POCE’s antioxidant activity as well as the transcript accumulation for an array of selected genes involved in the function of the stratum corneum and the epidermal barrier.

## Results

### Initiation and growth of callus cell lines

**Figure 1:**
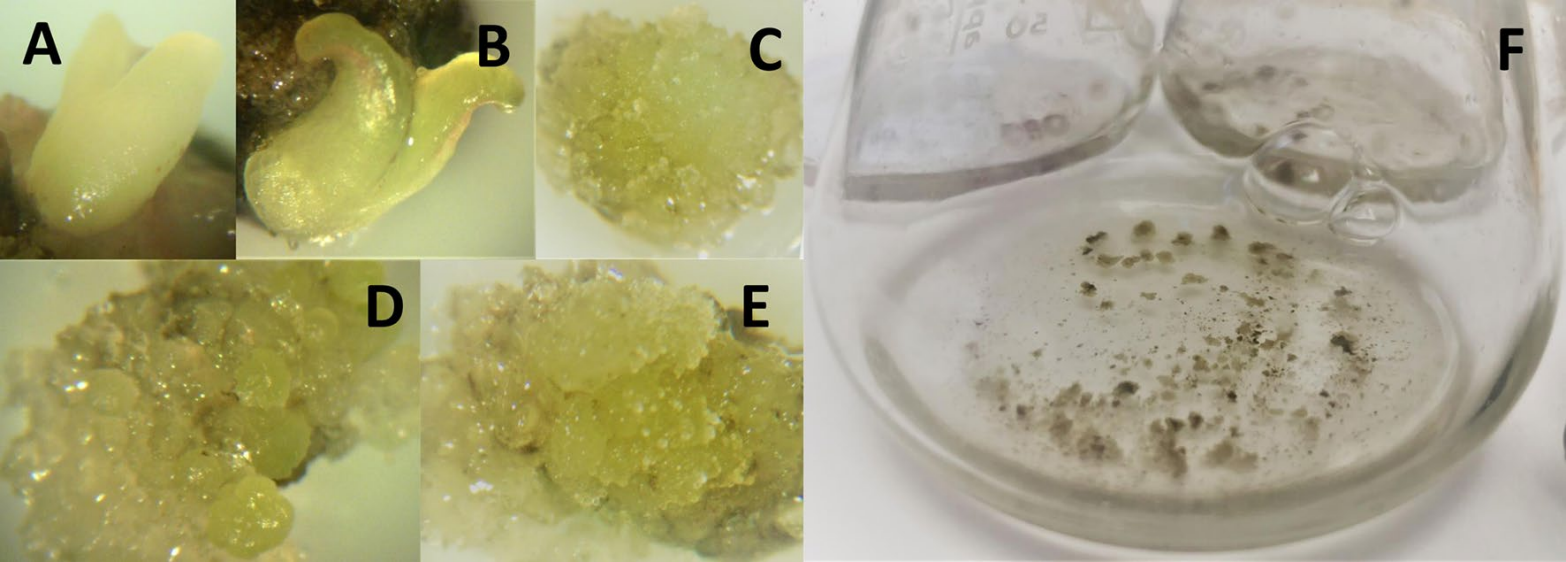
Initiation and growth of callus cell lines. Embryo formation (**A**, **B**), callus formation (**C**, **D**), callus with first stage globular embryos (**E**) growth of callus as small scale suspension cultures (**F**).

From the dissected embryos and endosperm tissue we obtained formation of embryos and callus (Fig. 1). For callus induction and growth, we compared several different medium combinations for their ability to induce callus from dissected embryos. Callus cultures were successfully developed, where the best callus induction and callus growth was obtained on MS basal medium supplemented with PGR3 in combination with PGR1. The formed callus cultures were grown on both solid and liquid medium. Elicitation of bioactive substances were performed for suspension cultures using elicitor (E1). Treatment with E1 resulted in increased production of metabolites and was therefore selected for all further work.

### Analysis of bioactive substances

The chemical analysis was based on total phenolic content (TPC), antioxidant (DPPH-EC50) and scavenging (E%) activity. For the elicitated cells we obtained a mean TPC values of 106.2 mg GA equivalent/g dry extract. The antioxidant activity (EC50) obtained for elicitated cells was 0.011 mg/mL, which corresponds to mg of extract required for a 50% decrease in absorbance of a 1 mL of DPPH solution. The scavenging activity (E%) for the extract concentration 1 µg/mL was 11% as compared to standard ascorbic acid in the same concentration (14%).

### HPLC analysis

**Figure 2:**
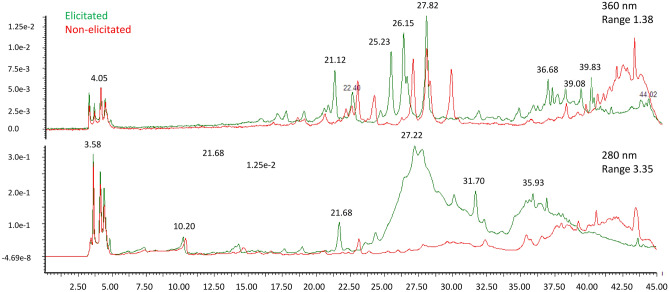
Represented chromatograms at 280 and 360 nm for elicitated (green) and unelicitated (red) cells.

A general HPLC analysis (TIC profiling) was also performed. The chromatograms showed that the elicitated cells, marked green in Fig. 2, exhibited an interesting complex chemical profile with several dominant peaks. We found that the elicitated cells showed a more complex profile with additional peaks at 280 and 320 nm as compared to unelicitated cells. The TIC profile between different analysed samples showed reproducibility and we were able to detect major peaks for RF = 9.28; RF = 19.6; RF = 28.03; RF = 34.87; RF = 42.72 at wavelength 280 nm and for RF = 18.88; RF = 22.33; RF = 23.22; RF = 24.70; RF = 30.38 at wavelength 360 nm.

### Cell viability assessment

ATP has been used as a tool for determining the functional integrity of living cells. To this context, we determined intracellular levels of ATP in NHEK cells incubated with different concentrations of POCE (Table 1). We observed that NHEK cells incubated with the lower concentrations of POCE exhibited significantly higher ATP levels compared to control (Table 1).

**Table 1:** Relative ATP levels (LUs) of NHEK cells after 48 h incubation with four concentrations of POCE.

Cells treatment	ATP levels (LUs)	Significance compared to control
Untreated NHEK (control)	6.37 E + 06 ± 1.22 E + 05	NS
*POCE* (1 μg/mL) treated NHEK	6.20 E + 06 ± 1.41 E + 05	NS
*POCE* (0.5 μg/mL) treated NHEK	5.97 E + 06 ± 1.21 E + 05	NS
*POCE* (0.1 μg/ml) treated NHEK	10.02 E + 06 ± 1.22 E + 05	< 0.05
*POCE* (0.05 μg/mL) treated NHEK	15.02 E + 06 ± 1.22 E + 05	< 0.05

Data are expressed as the mean ± SEM for eight replicates.

#### Mitochodria functionality

**Figure 3:**
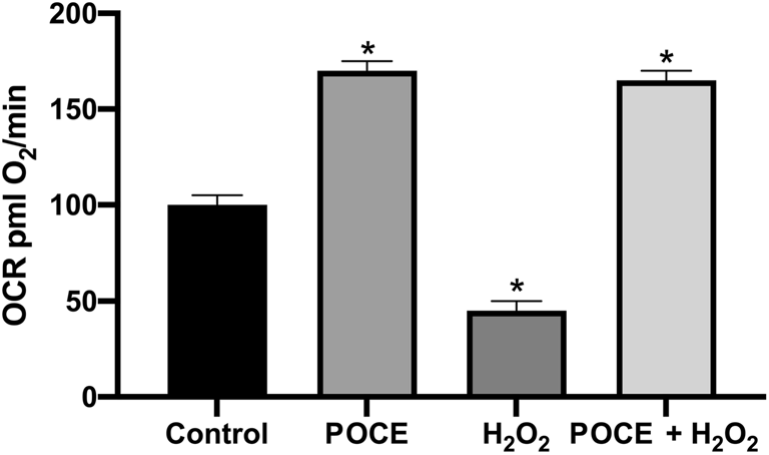
OCR levels expressed as mean ± SEM for: control (untreated NHEK cells), NHEK cells treated with POCE (POCE), NHEK cells treated with H_2_O_2_ (H_2_O_2_) NHEK cells pre-treated with POCE and after with H_2_O_2_ (POCE + H_2_O_2_). *p < 0.05 significantly different from the control (ANOVA test).

Mitochondrial metabolism has traditionally been thought of as a source of cellular ROS production responsbible for many cellular processes. However, increased oxidative stress can hamper mitochondrial energy. Here, we report the protective role of POCE in mitochondrial bioenergy against oxidative stress. We used hydrogen peroxidase as a stressor. Figure 3 depicts the protective role of POCE in increased oxidative stress in NHEK cells. Specifically, the OCR levels are significantly decreased under oxidative stress (H_2_O_2_) while the addition of POCE under or no oxidative stress increase the OCR levels.

#### Reconstructed human skin model

**Figure 4:**
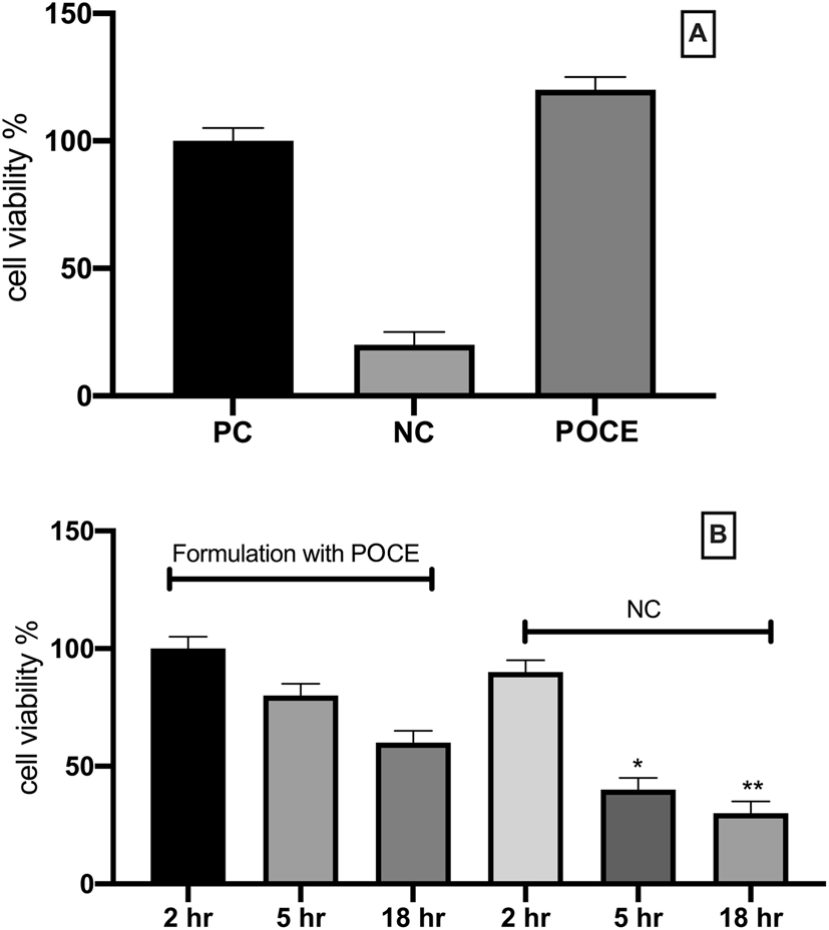
Cell viability levels expressed as mean ± SEM based on reconstructed human skin model for: (**A**) reconstructed human skin model treated with POCE, phosphate-buffered saline (DPBS) (positive control, PC) and 5% Sodium Dodecyl Sulfate (SDS) (negative control, NC). (**B**) reconstructed human skin model treated with a cosmetic formulation with POCE and 1% triton-100 as negative control (NC). *,**p < 0.05 significantly different from the formulation point.

To assess skin irritation potency conferred by POCE or a formulation contains POCE a reconstructed human epidermal model, EpiDerm EPI-200 (MatTek Corporation (Ashland, MA, USA) was used. According to Fig. 4, both the *Paeonia officinalis* var. *mascula* callus extract (Fig. 4A) and the formulation that contains it (Fig. 4B) were proven nonirritant for the human epidermis.

### Skin-related genes expression analysis

**Figure 5:**
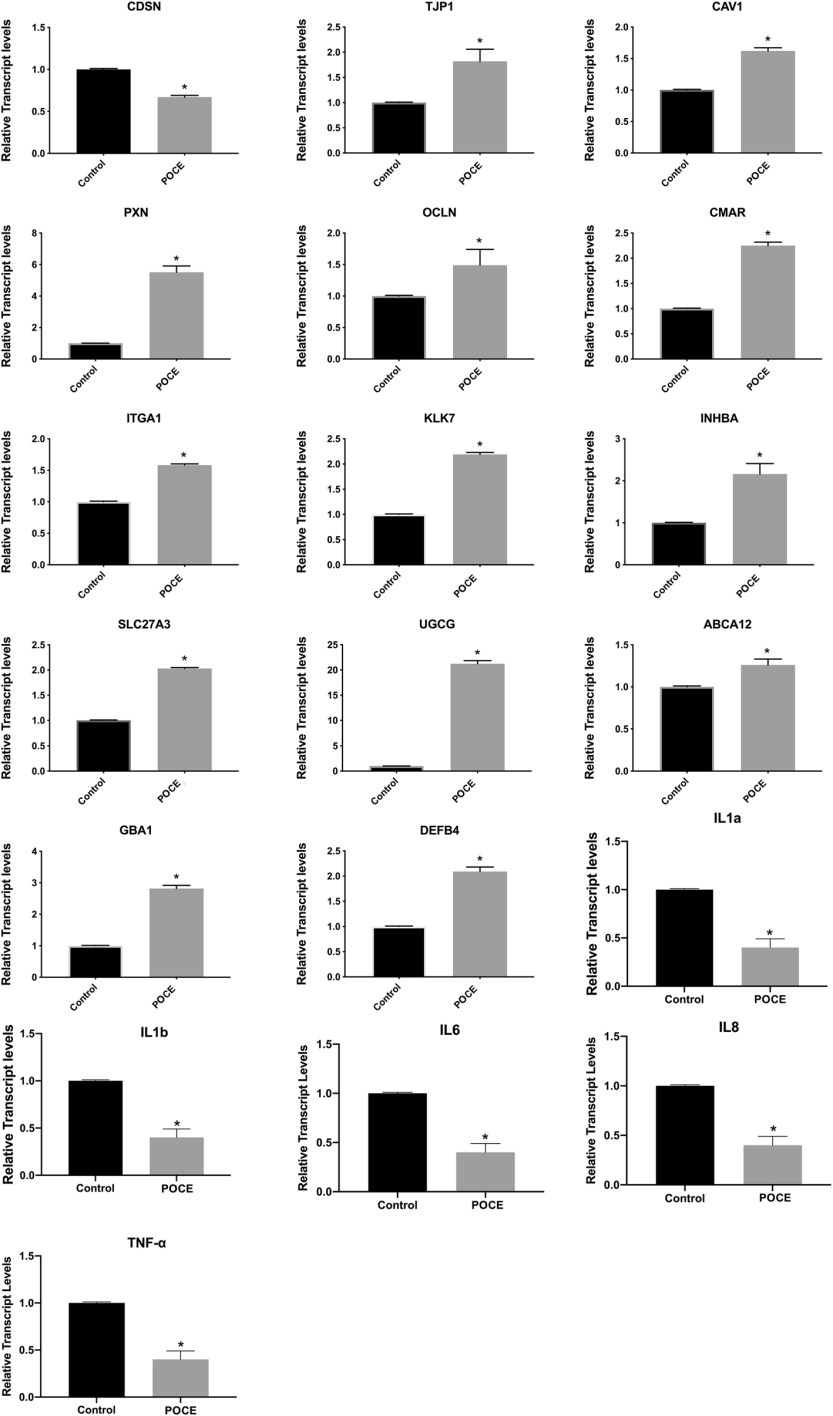
Relative gene expression of: CDSN, TJP1, CAV1, PXN, OCLN, CMAR, ITGA1, KLK7, INHBA, SLC27A3, UGCG, ABCA12, GBA1, DEFB4, IL1α, IL1β, IL6,IL8,TNF-α expressed as a fold change ± SEM compared to the control NHEK cells and using ACTB and GADPH as internal reference genes. The experimental conditions were control cells, cells treated with POCE (0.05 μg/ml) (POCE). *p < 0.05 indicates groups significantly different from the control (ANOVA test). The data correspond to the mean ± SEM of three independent experiments and six replicates each time.

To have an insight into the effect and molecular mechanism of POCE bioactivity in NHEK, we determined the expression of an array of genes involved in cell adhesion, desquamation, cell proliferation, lipids metabolism, and immune response. A detailed list of the target genes included in the present study and their respective relative expression levels is presented in Supplemental Table S1. Gene expression were measured by quantitative real time RT-PCR (RT-qPCR) using a developed platform containing the respective gene-specific primer pairs (Supplemental Table S2). Figure 5 shows the expression of genes that was significantly changed with the addition of *P. officinalis* var. *mascula extract.* As showed in Fig. 5, the expression of genes, inhibin subunit beta A (INHBA), Integrin alpha 1 (ITGA1), Occluding (OCLN), Paxillin (PXN), Caveolin 1 (CAV1), Tight junctions protein1 (TJP1), Cell Matrix adhesion regulator (CMAR), SCCE-stratum corneum chymotryptic enzyme (KLK7), FATPs-Fatty Acid Transport Proteins-3 (SLC27A3), ABCA-12 ATP-binding cassette A12 (ABCA12), b-glycocerebrosidase (GBA1) and β-defensins 4 (DEFB4) was up-regulated compared to control (p < 0.05). In contrast, the expression of Corneodesmosin (CDSN), interleukin-1 alpha (IL1a) and interleukin-1 beta (IL1b), interleukin-6 (IL-6), interleukin-8 (IL-8), tumor necrosis factor alpha (TNF-α) were down-regulated as compared to control (p < 0.05).

## Discussion

In this study, we have developed processes callus induction, growth and induction of bioactive substances for *P. officinalis* var. *mascula*. The formed callus showed a high scavenging activity, was high in total phenolic content and showed an interesting metabolic profile. For the formed callus we obtained a total phenolic content of 106.2 mg GA equivalent/g dry extract and EC50 values of 0.011 mg/mL. This is higher as compared to what has previously been recorded for *Paeonia suffruticosa* total phenolics in petals (51.7 mg GAE/g) and stamens (77.3 mg GAE/g), whereas flowers and buds showed higher total phenolic values (113.8 and 191.8 mg GAE/g, respectively)^2^. The DPPH radical scavenging activity was stronger for our developed callus culture as compared to all measured origins for *P. suffruticosa*, where the recorded EC50 values for petals, stamens, flowers and buds were 0.064, 0.042, 0.015 and 0.034 mg/mL, respectively. When comparing to callus cultures from other species, our developed *P. officinalis* callus showed higher total phenolic content and stronger DPPH radical scavenging activity as compared with for example *Zingiber officinale* Rosc. (45.9 mg GAE/g and 0.727 mg/mL), *Trifolium pretense* (40.8 mg GAE/g dry sample and 0.121 mg/mL) and *Asystasia gangetica* (27 mg GAE/g extract and 0.0868 mg/mL)^11–13^.

In addition, we evaluated the in vitro biological effects of *P. officinalis* var. *mascula* extract in human primary keratinocytes, to address its potential applications in skin related research. Interestingly, our results demonstrated that POCE increased the viability of human epidermal keratinocytes while enhancing mitochondrial activity under oxidative stress. The observed increased of ATP intracellular levels in keratinocytes clearly demonstrates that, at least in vitro, POCE enhances cell viability, cell proliferation and energy metabolism of keratinocytes. According to previous reports, this increase is associated with higher levels of mitochondrial activity, energy metabolism and cell proliferation^14,15^ and is indicative of a lack of cytotoxicity^14^ In line with this, POCE confers mitochondrial functionality under oxidative stress which is associated with the attenuated aging process as well as with the maintenance of significant cellular functions^16,17^.

To have broader spectrum of *POCE* action in NHEK cells we assessed genes involved in different pathways related to epidermis-related cellular function. Under our experimental conditions several genes where found to be transcriptionally modulated by POCE. To start with, we assessed the cell proliferation. The gene *INHBA* encodes a member of the TGF-beta (transforming growth factor-beta) superfamily of proteins involved in cell proliferation processes such as wound healing, inflammation^18,19^. In our study we observed that the gene expression of *INHBA* was up-regulated in NHEK cells treated with POCE compared to control. This induction is in accordance with a previous reported study^20^ and shows that POCE stimulates the proliferation of human keratinocytes confirming the previously mentioned increased ATP intracellular levels. Moreover, we explored the potential role of POCE in inflammation investigating the proinflammatory cytokines *IL1, IL6, IL8, TNF-α*. In particular, *IL1* promotes local inflammation and coagulation^21^, *IL6 *stimulates the synthesis of fibrinogen that ultimately contribute to inflammatory acute phase^21^ while *IL8* is a involved in many inflammation related processes such as chemotaxis of basophil and angiogenesis^22^ and *TNF-α* is an essential mediator in inflammation^23^. Here, we observed that the gene expression of *IL1*, *IL6, IL8, TNF-α* were downregulated in NHEK cells treated with POCE compared to control. This downregulation is in agreement with previous reports^24–26^ emphasizing POCE’s role as an anti-inflammatory agent. On the other hand, we investigated genes that are involved in immune response in epidermis layer. Human β-defensin 2 (hBD2), encoded by the *DEFB4* gene, is an inducible antimicrobial peptide with molecular mass of 4–6 kDa acting as an endogenous antibiotic in the defense of host against Gram-positive and Gram-negative bacteria, fungi and the envelope of some viruses, and is involved in the innate immune response because its release is induced by pro-inflammatory cytokines, endogenous stimuli, infections or wounds^27,28^. Interestingly, in our study, we observed an induction of *DEFB4* in NHEK cells treated with POCE. The same induction has been observed before in human primary keratinocytes^24–26,29^, although this trend suggests a potential role of POCE in antimicrobial process regulation yet it needs to be further elucidated.

Cell adhesion is not only essential for all multicellular organisms to interact and coordinate within cell populations but also critical for unicellular organisms to exchange signals with their microenvironment. Here, we assessed the gene expression of *ITGA1, OCLN, PXN, CAV1, TJP1, CMAR* that are related to cell adhesion and epidermal barrier function. Adhesion receptors include integrins, the immunoglobulin superfamily, cadherins, cellular adhesion molecules (CAMs), and homing receptors. Among all the cell adhesion receptors, integrins are important for “maintaining the integrity of the cytoskeletal-ECM linkage^30^. ITGA1 Integrin alpha-1/beta-1 is a receptor for laminin and collagen^31^. We observed that the gene expression of *ITGA1* was up-regulated in NHEK treated with POCE as compared to control, which potentially indicate an enhancement of integrins activity. Moreover, we evaluated the genes that encode the epithelial TJ proteins (occludin, and TJP1). *OCLN* encodes occludin, one of the epidermal tight junctions, since the expression of *OCLN* restores the function of tight junctions in disturbed epithelial cells^32^ it indicates that the increase of *OCLN* expression will restore skin barrier function according to previous report^33^. TJP1 is found in cytoplasmic plaques of tight junctions and is thought that *TJP1* is involved in creating the proper organization of proteins within the tight junctional plaque^34^. Previous study showed that improving the expressions of tight junction proteins such as occluding or *TJP1* has an effect on repairing epithelial barrier either in vitro and in vivo^35^. In our study, both the expression of *OCLN* and *TJP1* were upregulated in NHEK treated with POCE showing the positive function of cytoplasmic plaques of tight junctions due to the addition of the extract. The paxillin (*PXN*) gene is a 68-kDa tyrosine-containing protein that acts as an adaptor protein that integrates integrin, adhesion molecule, and growth factor signals^36^. *PXN* interacts with integrins during matrix organization and tissue remodeling by destabilizing focal adhesions, such as the linkage between *PXN* and actin filaments^37^. Caveolae are sphingolipid- and cholesterol-rich plasma membrane microdomains found in diverse cell types, most prominently in adipocytes, endothelial cells, fibroblasts, and muscle cells^38^. Caveolae-1 has a critical role in lipids uptake of these cells^39^. It has been reported that induction of *CAV-1* is associated with keratinocytes differentiation^38^. In our study, we observed that the gene expression of *CAV-1* and *PXN* were up-regulated in NHEK treated with POCE potentially indicating keratinocytes differentiation. The gene *CMAR* encodes a mitochondrial metalloprotease protein that is a member of the AAA family. *CMAR* has been suggested to be a signal transduction molecule influencing cell adhesion to collagen. Here, the expression of *CMAR* was up-regulated in NHEK treated with POCE compared to control. However, further work is needed to fully understand this interaction.

Another process that is essential in skin barrier function is desquamation process. In the desquamation process, kallikrein (KLK)-related peptidases such as *KLK5* and *KLK7* degrade *CDSN* and induce cleavage of corneodesmosomes, resulting in detachment of corneocytes^40^. In the SC, the corneocytes are tightly bound together via corneodesmosomes, the end product of epidermal desmosomes modified by the incorporation of corneodesmosin (CDSN). It has been reported that down-regulated levels of *CDSN* are associated with barrier-related defects^41^. In our study we observed a down-regulation in expression levels of *CDSN* in NHEK treated with POCE compared to control. Nevertheless, further work is needed to fully understand this interaction. On the other hand, *KLK7* has a primary role in the physical and biochemical barrier functions of the stratum corneum^42^. Recently it has been reported that reduction of *KLK7* is associated with unhealthy skin states^43^ interestingly, in our study we observed that the expression of *KLK7* was up-regulated in NHEK treated with POCE indicating a protective activity of stratum corneum functions.

Furthermore, we investigated the lipids replenish system. The gene *SLC27A3* belongs to the *SLC27* gene family that is comprised of six members, *SLC27A1-6*, which encode fatty acid transporters proteins (FATPs)^44^. The FATPs family play a vital role in formation of the epidermal permeability barrier^45^. It has been reported that reduced levels of *SLC27A3* are connected with skin dysfunction and skin dehydration^46^. In our study we observed that the expression of *SLC27A3* was up-regulated in NHEK treated with POCE, indicating an enhancement of epidermal barrier preventing dehydration. The gene *UGCG* encodes the enzyme which catalyze the first glycosylation step in the biosynthesis of glycosphingolipids^47^. Previous study showed that *UGCG* deficiency in skin leads to epidermal barrier dysfunction. Epidermal barrier function has mainly been attributed to the SC, composed of lipid-embedded corneocytes and a lipid-enriched extracellular matrix^48^, as well as to TJ proteins preventing paracellular diffusion and transport^49^. Disturbance of one of those factors may lead to severe dermatoses such as ichthyoses, atopic dermatitis and psoriasis^50–54^. Here, we observed that the expression of *UGCG* was up-regulated in NHEK cells treated POCE, which indicates that the extract might support optimum epidermal barrier function. The *ABCA12* encodes a transporter of lipids that delivers glucosylceramide to epidermal lamellar bodies in keratinocytes^55^. The increase of *ABCA12* expression is in accordance with previous report^33^ where it’s stated that increase of *ABCA12* expression would restore the delivery of complex sphingolipids to the lamellar bodies of keratinocytes and accelerate maturation of the skin permeability barrier function. GBA1 is an acid β-glucosidase normally located in lysosomes, converts (glucosyl) ceramides into ceramides, which is crucial to generate an optimal barrier function of the outermost skin layer, the stratum corneum (SC)^56,57^. Previous reports have showed that reduction on *GBA1* expression may lead to an impaired barrier function that has been encountered in several inflammatory skin diseases^58,59^. Here, we observed that the expression of *GBA1* was up-regulated in NHEK treated with POCE indicating an enhancement of the optimal barrier function of the SC.

Additionally, the reconstituted human skin model was utilized to further confirm the protective properties of POCE on human epidermis^60,61^. In our study, both the POCE and its incorporation in a cosmetic formulation were proven non irritant for the epidermis which is an essential step during the development of new skin care related products.

To summarize, in the present study, we developed an effective callus extract independently of external factors (e.g. soil composition or climate, microorganisms or insects) preserving the uniqueness of the plant species *Paeonia officinalis* var. *mascula*. We demonstrated that the *P. officinalis* var. *mascula* callus extract confers mitochondrial activity and modulates the expression levels of several genes involved in epidermis function. In line with that, we proved the efficacy of this extract on a human reconstructed skin model. Future studies are required to shed more light into elucidating the molecular mechanism(s) underlying the cytoprotective properties of *P. officinalis* var. *mascula* as well as its efficiency at clinical level.

## Methods

### Callus induction and growth of callus

During the spring 2018 plant material from *P. officinalis* var. *mascula* was collected and used to initiate the in vitro cultures. The material was surface sterilised using a 1:50 dilution of Clorox solution and thereafter rinsed using sterile water. Seeds were used to initiate in vitro callus cultures; these were grown on MS^62^ basal medium supplemented with BAP (benzylaminopurine, Duchefa Biochemie B.V, Haarlem, Netherlands) and gibberellic acid (GA3, Duchefa Biochemie B.V, Haarlem, Netherlands). The dissected embryos were used for induction and growth of callus cultures. For callus induction and growth MS basal medium supplemented with different combinations of PGR1-3 were compared. Callus was subcultured every third week on MS basal medium supplemented with PGR1 and 3. For elicitation Elicitor 1 was used.

### Extraction of bioactive substances

Bioactive substances were extracted using a multistep extraction process: water, followed gradually increasing concentrations of ethanol, ranging from 50 to 80%, to obtain extracts a broad range of active substances with different chemical and polar properties. Biomass:solvent ratios of 1:30–1:50 was used. Extraction was performed using ultrasonic water bath, 3 × 10 min cycles, followed by centrifugation, removal of supernatant and filtration.

### Total phenolic content

Total phenolic content was measured using the Folin-Ciocalteu reagent (FCR assay), according to Chakrabothy and Ghorpade^63^ with the following modifications. A total of 20 µL extract was mixed with 780 µL H_2_Odd and 50 µL of Folin-Ciocalteu reagent (EMD Millipore, Burlington, MA, USA). After 7 min, 150 µL of saturated Na_2_CO_3_ solution (VWR Chemicals, Radnor, PA, USA) was added. The samples were mixed and incubated at 30 °C for 1 h in full darkness. Absorbance was thereafter measured using spectrophotometric measurements at 765 nm (GENESYS 20, Thermo Scientific, Waltham, MA, USA). Gallic acid (Cayman Chemical Company, Ann Arbor, MI, USA) was used as a standard and the calibration curve was linear in the range 0.03–1 mg/mL. The results were presented as mg gallic acid equivalent (GAE)/g dry extract. Two biological replicates were analysed, where each sample was examined for three different extract dilutions, each of them in three technical replicates.

### Scavenging capacity by DPPH assay

Radical scavenging activity of the crude extracts were determined using the 2,2-diphenyl-1-picrylhydrazyle (DPPH) assay as described by Brand-Williams et al.^64^,^65^ with the following modifications. The reaction was downscaled to 1 mL total volume. A total of 25 µL extract was mixed with 975 µL of a 24 µg/mL (w/v) DPPH solution in ethanol (SIGMA, Saint Louis, MO, USA; VWR Chemicals, Radnor, PA, USA) and incubated at room temperature for 30 min in full darkness. Ascorbic acid (Duchefa Biochemie, Haarlem, The Netherlands) was used as a standard and the calibration curve was linear in range 0.00003–0.004 mg/mL. The absorbance was measured using spectrophotometric measurements at 515 nm (GENESYS 20, Thermo Scientific, Waltham, MA, USA). Two biological replicates were analysed, where each sample was analysed for six different extract concentrations. The measurement was performed using two technical replicates. The results were presented as EC50 value [mg dry extract/mL DPPH solution].

### HPLC analysis

For HPLC analysis, the Waters Alliance 2790 HPLC Separations Module with 996 PDA Detector, with Column Hypersil GOLD instrument (RP, C18, dimensions 250 × 4.6 mm, particle size of 5 µm) (Thermo Scientific, Waltham, MA, USA) was used. A total of 10 µL of a 10 mg/mL extract was injected and analysed. The mobile phase consisted of A: 1% acetic acid (VWR International, Radnor, PA, USA) and B: methanol (VWR Chemicals, Radnor, PA, USA) with gradient (Table 2) and a flow of 1 mL/min.

**Table 2:** Gradient for HPLC analysis.

Time (min)	% mobile phase A	% mobile phase B
0	95	5
2	95	5
10	75	25
20	60	40
30	50	50
40	0	100
45	95	5

*Mobile phase A* 1% acetic acid, *Mobile phase B* methanol.

### Human skin cell culture

Primary Normal Human Epidermal Keratinocytes (NHEK) isolated from normal human adult skin were purchased from Lonza Clonetics (Lonza Walkersville, USA)^17^. Cells were cultured according to Lonza instructions. The cells were grown in a recommended media FGM-2 BulletKit that contained 2% serum. Cells were subcultured when reached 70–80% confluence. The cells were incubated for 48 h with POCE in different concentrations (0.05–1 μg/ml). After the incubation, ATP determination and RNA isolation followed.

### Determination of ATP levels

*The* determination *intracellular* ATP was based on ViaLight HS BioAssay kit (Lonza)^66,67^. GloMax 20/20 single-tube luminometer (Promega) was used.

### Mitochondria functionality

Oxygen consumption rate (OCR) in NHEK cells was measured in real-time using a Seahorse XFe24 Analyzer (Agilent, USA). Cells (2 × 10^4^ cells/well) were seeded in the XF-24 plate and incubated for 48 h with the POCE extract (0.050 μg/mL). After incubation, cells were exposed to H_2_O_2_ (0.5 mM) for 1 h, according to previously conducted studies to determine the concentration of H_2_O_2_ as well as the exposure time^66,68^. The OCR levels in control (untreated NHEK cells), POCE pre-treated NHEK cells and in cells stressed with H_2_O_2_ with and without pretreatment with the POCE were measured. The readings were performed three times for each state with three min given for each reading and data were expressed as pmol of O_2_ consumed per minute.

### Gene expression characterization by RT-qPCR

At first place, total RNA (tRNA) (500 ng) was isolated from all the samples using a Nucleospin RNA kit (Macherey–Nagel, Germany) and cDNA was prepared using the PrimeScript-RT reagent kit (Takara Bio, Japan) following the manufacturer’s instructions. RT-qPCR was used to characterize the molecular changes associated with POCE bioactivity in NHEK cells as described before^66–68^. Briefly, quantitative RT-PCR reactions were performed using KAPA SYBR FAST qPCR Master Mix (Kapa Biosystems, Inc., Wilmington, Massachusetts, US), gene specific primers at a final concentration of 0.5 μΜ each and 1 μL of the cDNA as template. The RT-qPCR cycle was performed in a CFX connect TM Real Time System (Bio-Rad Laboratories, Hercules, California, USA) and the cycle used was: 95 °C for 10 min, followed by 40 cycles of 95 °C for 15 s and 60 °C for 1 min. Supplementary Tables S1 and S2 provide a list of target genes as well as the respective gene-specific primer pairs respectively. The primer pairs were designed using Primer Express 1.5 software (Applied Biosystems, Darmstadt, Germany). The primer specificity and formation of primer-dimers was monitored by dissociation curve analysis and gel electrophoresis of the reaction products on a 4% (w/v) TBE agarose gel. For the relative gene expression, comparative threshold cycle (Ct) method was used^69^ and normalization based on two reference genes Actin beta (ACTB) and Glyceraldehyde-3-phosphate dehydrogenase (GAPDH). The experimental conditions that were analyzed by RT-qPCR and presented here, were: untreated NHEK cells (control), NHEK cells treated with *POCE* (0.05 μg/mL) (*POCE* 0.05 μg/mL). All RT-qPCR reactions were performed on three biological replicates following by eight technical repeats.

#### Reconstructed human skin model

To assess of any skin irritation potency conferred by POCE a reconstructed human epidermal model, EpiDerm EPI-200 (MatTek Corporation Ashland, MA, USA) was used. The model consists of normal, human-derived epidermal keratinocytes, which have been cultured to form a multi-layered, highly differentiated model of the human epidermis. The tissue exhibits organised basal, spinous and granular layers, and a multi-layered stratum corneum containing intercellular lamellar lipid layers arranged in patterns analogous to those found in vivo^61^. Because it closely mimics the human epidermis, it has proven its scientific relevancy in studies where dermal exposure and toxicity is anticipated: skin corrosion testing of chemicals, skin irritation studies of cosmetic products and raw materials, phototoxicity, and skin penetration studies^60,70^. Cell viability was measured by the MTT reduction assay according to manufacturer’s instructions. As positive and negative control for skin irritation, Dulbecco’s phosphate-buffered saline (DPBS) and 5% Sodium Dodecyl Sulfate (SDS) were used, respectively. Moreover, we evaluated the potential skin irritation of cosmetic formulation which contains POCE using the same reconstructed human skin model. The cosmetic formulation was an oil in water basic cream base with POCE incorporated (Supplemental Table S3). The parameters measured in vitro were the percentage cell viability using an the MTT Effective Time 50 (ET50) assay after topical application of the products for different exposure times (2 h, 5 h and 18 h) according to manufacturer’s instructions. For negative control, 1.0% Triton X-100 was used.

### Statistical analysis

All the results are presented as mean ± SEM. One-way ANOVA following by Bonferroni’s multiple corrections test was used to detect statistical differences as it has been reported before^66–68^. All data were normally distributed (Kolmogorov–Smirnov test). The level of significance was p < 0.05. The statistical analysis was accomplished using SPSS 17.0 (SPSS Inc., Chicago, IL, USA).

## Supplementary information

Supplementary Tables.
