# Author Correction: In vitro protective effects of *Paeonia officinalis* var. *mascula* callus extract on human keratinocytes

**DOI:** 10.1038/s41598-023-34609-7

**Published:** 2023-05-19

**Authors:** Sophia Letsiou, Artemis Bakea, Anna Holefors, Jadwiga Rembiesa, Eleni Spanidi, Konstantinos Gardikis

**Affiliations:** 1 Laboratory of Biochemistry, Research and Development Department, APIVITA S.A., Industrial Park of Markopoulo Mesogaias, Markopoulo Attiki, 19003 Athens, Greece; 2 In Vitro Plant-Tech AB, Geijersg 4B, 21618 Limhamn, Sweden

Correction to: *Scientific Reports* 10.1038/s41598-020-76169-0, published online 05 November 2020

The original version of this Article contained errors.

Firstly, Eleni Spanidi and Konstantinos Gardikis were omitted from the author list in the original version of this Article.

The Author Contributions section now reads:

“S.L. conceived, planned, and oversaw the experiments. A.B. carried out the experiments on the RT-qPCR analysis. A.H. and J.R. initiated the callus cell line and carried out experiments on the antioxidant assay and on HPLC analysis. S.L. analyzed and integrated the datasets and drafted the manuscript. K.G. conceived the project. K.G. revised the manuscript. E.S. collected the material and carried out experiments on antioxidant assays. All authors critically read and contributed to improving the MS.”

Secondly, the Acknowledgements section of this Article: “This study was made possible thanks to financial support provided by APIVITA SA.” has been removed.

Lastly, two synonymous names have been used in the paper to describe the studied species. This has been corrected for consistency, and “*Paeonia officinalis* var. *mascula*” (and the corresponding abbreviation “POCE”) is now used throughout the article. Additionally, sentences implying potential application of the plant extract in cosmetics have been removed.

The original Article and accompanying Supplementary Information file have been corrected.

