# Supplementary material for: In vitro protective effects of Paeonia officinalis var. mascula callus extract on human keratinocytes
Source: Sci Rep. 2020 Nov 5;10:19213. doi: 10.1038/s41598-020-76169-0 (PMC7645794; doi:10.1038/s41598-020-76169-0)
Supplement: Supplementary file 1 — Supplementary Tables. [file 41598_2020_76169_MOESM1_ESM.docx]

*In vitro* protective effects of Paeonia officinalis var. mascula callus extract on human keratinocytes.

Sophia Letsiou^*1^, Artemis Bakea^1^, Anna Holefors^2^, Jadwiga Rembiesa^2^, Eleni Spanidi^1^, Konstantinos Gardikis^1^.

1. Laboratory of Biochemistry, Research and Development Department, APIVITA S.A., Industrial Park of Markopoulo Mesogaias, 19003 Markopoulo Attiki, Athens, Greece.

2. In vitro Plant-tech AB, Geijersg 4B, SE-21618 Limhamn, Sweden

*Correspondence: Sophia Letsiou sletsiou@gmail.com, Laboratory of Biochemistry, Research and Development Department, APIVITA SA, Industrial Park of Markopoulo Mesogaias, 19003, Markopoulo Attikis, Athens, Greece. sletsiou@gmail.com, Tel: 0030 2102854428, Fax: +30 2102843580.

Table S1. Relative mRNA expression of genes.

| Gene | Gene ID | Relative mRNA expression ration versus control NHEK treated with POCE (0.05μg/ml) | Pvalue  (ANOVA) |
| --- | --- | --- | --- |
| INHBA | 3624 | 2.172 ± 0.001 | P <0.05 |
| ITGA (ITGA1) | 3672 | 1.612 ± 0.001 | P <0.05 |
| OCLN | 100506658 | 1.386 ± 0.001 | P <0.05 |
| PXN | 5829 | 4.512 ± 0.098 | P <0.001 |
| CAV1 | 857 | 1.531 ± 0.001 | P <0.05 |
| TJP1 | 7082 | 1.985 ± 0.001 | P <0.05 |
| CMAR( SPG7) | 6687 | 2.312 ± 0.001 | P <0.05 |
| KLK7 | 5650 | 2.031 ± 0.001 | P <0.05 |
| SLC27A3 | 11000 | 2.012 ± 0.001 | P <0.05 |
| UGCG | 7357 | 20.01 ± 0.912 | P <0.001 |
| ABCA12 | 26154 | 1.389 ± 0.001 | P <0.05 |
| GBA1 | 2629 | 2.512 ± 0.001 | P <0.05 |
| DEFB4 | 56519 | 2.012 ± 0.001 | P <0.05 |
| CDSN | 1041 | 0.632 ± 0.001 | P <0.05 |
| IL-1a | 3552 | 0.468 ± 0.001 | P <0.05 |
| IL-1β | 3553 | 0.492 ± 0.001 | P <0.05 |
| IL-6 | 3569 | 0.412 ± 0.001 | P <0.05 |
| IL-8 | 3576 | 0.462 ± 0.001 | P <0.05 |
| TNF-α | 7124 | 0.432 ± 0.001 | P <0.05 |

Table S2. Gene name, Accesion No, Kegg pathway, Primer

| **Gene Symbol** | **Gene Name** | **Accession No** | **Kegg pathway** | **Primmer F (5'-3')** | **Primmer R (5'-3')** |
| --- | --- | --- | --- | --- | --- |
| INHBA | inhibin subunit beta A | [NM_002192.4](https://www.ncbi.nlm.nih.gov/nuccore/NM_002192.4) | [map04350](https://www.genome.jp/dbget-bin/www_bget?pathway:map04350) | GCAGAAGTCTGGCTCTTCCTAA | CCCCTCCTCTTCTTTCTTCTTC |
| ITGA 1 | Integrin alpha1 | [NM_181501.2](https://www.ncbi.nlm.nih.gov/nuccore/NM_181501.2) | [map04810](https://www.genome.jp/dbget-bin/www_bget?pathway:map04810) | GAAGAACCTCCTGAAACCCTTT | TGATGTCATATTGGGGAATGAA |
| OCLN | Occluding | [NM_001205254.2](https://www.ncbi.nlm.nih.gov/nuccore/NM_001205254.2) | [map04670](https://www.genome.jp/dbget-bin/www_bget?pathway:map04670) | TTTGCTGTGAAAACTCGAAGAA | CGCCAGTTGTGTAGTCTGTCTC |
| PXN | Paxillin | [NM_001080855.3](https://www.ncbi.nlm.nih.gov/nuccore/NM_001080855.3) | [map04370](https://www.genome.jp/dbget-bin/www_bget?pathway:map04370) | AGTGCCAAAACTTCCAGTGTCT | AGCTCTCCAGTTCATCCAAGAG |
| CAV1 | Caveolin 1 | [NM_001172895.1](https://www.ncbi.nlm.nih.gov/nuccore/NM_001172895.1) | [map05100](https://www.genome.jp/dbget-bin/www_bget?pathway:map05100) | CGTGGTCAAGATTGACTTTGAA | AGATGGAATAGACACGGCTGAT |
| TJP1 | Tight junction protein 1 | [NM_001301025.3](https://www.ncbi.nlm.nih.gov/nuccore/NM_001301025.3) | [map04530](https://www.genome.jp/dbget-bin/www_bget?pathway:map04530) | TACAATGGAAAACTGGGCTCTT | AAATGGTTACAGGCCTCAGAAA |
| CMAR(SPG7) | Cell Matrix adhesion regulator | [NM_001363850.1](https://www.ncbi.nlm.nih.gov/nuccore/NM_001363850.1) |  | GAATGCAGGTTGCAAATATTGA | TCTTTGAAGCTGACTCCTTTCC |
| KLK7 | kallikrein related peptidase 7 | [NM_001207053.2](https://www.ncbi.nlm.nih.gov/nuccore/NM_001207053.2) |  | ATCCATGGTGAAGAAAGTCAGG | TTGAGTGTAGACTCCTGGGTCA |
| SLC27A3 | solute carrier family 27 member 3 | [NM_001317929.4](https://www.ncbi.nlm.nih.gov/nuccore/NM_001317929.4) | [map04931](https://www.genome.jp/dbget-bin/www_bget?pathway:map04931) | AGAGTTTCTGTGGCTCTGGTTC | GGACACCACACAGCTGATAGAA |
| UGCG | UDP-glucose ceramide glycosyltransferase | [NM_003358.3](https://www.ncbi.nlm.nih.gov/nuccore/NM_003358.3) | [map00600](https://www.genome.jp/dbget-bin/www_bget?pathway:map00600) | TCCCAAATATGAAGTGCTCCTT | TTCCTGTCACACATTTGAAACC |
| ABCA12 | ATP- binding cassette A12 | [NM_015657.3](https://www.ncbi.nlm.nih.gov/nuccore/NM_015657.3) | [map02010](https://www.genome.jp/dbget-bin/www_bget?pathway:map02010) | TGCTTGGTATGTCAGGAATGTC | TATACAAAAATGGTGCCTGCTG |
| GBA | b-glycocerebrosidase | [NM_000157.4](https://www.ncbi.nlm.nih.gov/nuccore/NM_000157.4) | [map00600](https://www.genome.jp/dbget-bin/www_bget?pathway:map00600) | GCTCAAGATACCCCTGATTCAC | CGGACATTGTGGTGAGTACTGT |
| DEFB4 | β- defensins 4 | [NM_019728.4](https://www.ncbi.nlm.nih.gov/nuccore/NM_019728.4) | [map04621](https://www.genome.jp/dbget-bin/www_bget?pathway:map04621) | CTCCTCTTCTCGTTCCTCTTCA | TTCTAGGGCAAAAGACTGGATG |
| CDSN | Corneodesmosin | [NM_001264.5](https://www.ncbi.nlm.nih.gov/nuccore/NM_001264.5) |  | CTCTTCCTCTTCCCAGACCTTT | TGAAGTAGCCCACAGGGTAGAT |
| IL-1a | Interleukin 1 alpha | [NM_000575.5](https://www.ncbi.nlm.nih.gov/nuccore/NM_000575.5) | map04064 | CATGTCAAATTTCACTGCTTCATCC | GTCTCTGAATCAGAAATCCTTCTATC |
| IL-1β | Interleukin 1 beta | NM_000576.3 | map04064 | GCATCCAGCTACGAATCTCC | CCACATTCAGCACAGGACTC |
| IL-6 | Interleukin 6 | NM_000600.5 | map04064 | ATGAACTCCTTCTCCACAAGCGC | GAAGAGCCCTCAGGCTGGACTG |
| IL-8 | Interleukin 8 | NM_000584.4 | map04064 | ATGACTTCCAAGCTGGCCGTG | TGAATTCTCAGCCCTCTTCAAAAACTTCTC |
| TNF-α | Tumor necrosis factor At | NM_000594.4 | map04064 | CAGAGGGAAGAGTTCCCCAG | CCTTGGTCTGGTAGGAGACG |
| ACTB | actin, beta | [NM_003254.2](https://www.ncbi.nlm.nih.gov/nuccore/NM_003254.2) | [hsa04066](http://www.genome.jp/dbget-bin/www_bget?pathway:hsa04066) | TTTATCCATCCCCTGCAAACTG | TTTTCAGAGCCTTGGAGGAGC |
| GAPDH | glyceraldehyde-3-phosphate dehydrogenase | [NM_001101.3](http://www.ncbi.nlm.nih.gov/nuccore/NM_001101.3) | [hsa04810](http://www.genome.jp/dbget-bin/www_bget?pathway:hsa04810) | CTGTCCACCTTCCAGCAGATGT | AGCATTTGCGGTGGACGAT |

Table S3. Ingredients breakdown of a cosmetic formulation with POCE.

| **A/A** | **INCI (EU)** | **% (w/w)** |
| --- | --- | --- |
| 1 | Aqua | 71,9700 |
| 2 | Neopentyl Glycol Diheptanoate | 4,0800 |
| 3 | Dicaprylyl Ether | 3,5000 |
| 4 | Cetearyl Alcohol | 3,1350 |
| 5 | Glycerin | 3,0000 |
| 6 | Cocoglycerides | 3,0000 |
| 7 | Glyceryl Stearate | 2,0000 |
| 8 | Propylene glycol | 1,6500 |
| 9 | PEG-100 Stearate | 1,2000 |
| 10 | POCE | 1,0000 |
| 11 | PEG-20 Stearate | 0,6650 |
| 12 | Phenoxyethanol | 0,5550 |
| 13 | Tocopheryl Acetate | 0,5000 |
| 14 | Panthenol | 0,5000 |
| 15 | Sorbitan Stearate | 0,5000 |
| 16 | Caprylic/Capric Triglyceride | 0,3500 |
| 17 | Caprylyl Glycol | 0,4450 |
| 18 | Vitis vinifera leaf extract | 0,3500 |
| 19 | Ethylhexylglycerin | 0,3000 |
| 20 | Carbomer | 0,3000 |
| 21 | Allantoin | 0,2000 |
| 22 | Bisabolol | 0,2000 |
| 23 | Sodium Hydroxide | 0,2000 |
| 24 | Disodium EDTA | 0,1500 |
| 25 | Tocopherol | 0,1500 |
|  | **Total** | **100** |
